# Anti-microbial Effects In Vitro and In Vivo of *Alstonia scholaris*

**DOI:** 10.1007/s13659-020-00294-6

**Published:** 2021-01-03

**Authors:** Yun-Li Zhao, Zhong-Ping Gou, Jian-Hua Shang, Wan-Yi Li, Yu Kuang, Ming-Yuan Li, Xiao-Dong Luo

**Affiliations:** 1 State Key Laboratory of Phytochemistry and Plant Resources in West China, Kunming Institute of Botany, Chinese Academy of Sciences, Kunming, 650201 People’s Republic of China; 2 Key Laboratory of Medicinal Chemistry for Natural Resource, Ministry of Education; Yunnan Provincial Center for Research & Development of Natural Products; School of Chemical Science and Technology, Yunnan University, Kunming, 650091 P. R. China; 3 Institute of Drug Clinical Trials, West China Hospital, Sichuan University, Chengdu, 610041 China; 4 West China School of Basic Medical Sciences & Forensic Medicine, Sichuan University, Chengdu, 610041 China

**Keywords:** *Alstonia scholaris*, Total alkaloids, Acute respiratory infections, Anti-virus, Anti-bacteria

## Abstract

*Alstonia scholaris* could be used as a traditional medicinal plant in China for the treatment of acute respiratory, which might be caused by respiratory tract infections. The investigation tested the anti-infective effects of total alkaloids extract (TA) from leaves of *A. scholaris*, and as a result, TA inhibited herpes simplex virus type 1 (HSV-1), respiratory syncytial virus (RSV) and influenza A virus (H1N1) in vitro respectively. In addition, the survival days of mice were prolonged, and the lung weights and mortality of mice were decreased significantly, after oral administrated TA in H1N1 and beta-hemolytic *streptococcus* infectious models in vivo respectively. The finding supported partly the traditional usage of *A. scholaris* in the treatment of respiratory infections.

**Graphic Abstract:**

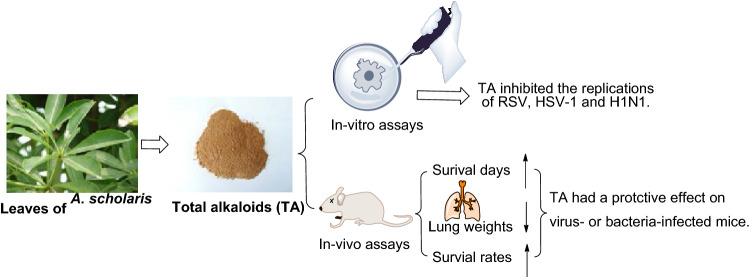

**Supplementary information:**

The online version of this article (10.1007/s13659-020-00294-6) contains supplementary material, which is available to authorized users.

## Introduction

Acute respiratory infections (ARIs) are the most common infections in humans, ranged from common cold through to very severe acute respiratory syndrome. ARIs are caused by over 200 viruses or bacteria [1]. Respiratory viruses seem to cause or serve as a co-pathogen in most cases of ARIs in epidemiologic studies. The specific viruses most frequently associated with acute bronchitis, in order of frequency of occurrence, are influenza, parainfluenza, respiratory syncytial virus (RSV), coronavirus, herpes simplex virus (HSV), adenovirus, and rhinoviruses [2]. The upper airway is a major ecological reservoir of bacterial species, bacterial infection ARIs approximately accounts for 15% of all patients, most of which are hemolytic *streptococcus*, followed by *haemophilus* influenzae, pneumococcus and *staphylococcus*, and occasionally gram-negative *bacill* [3].

Antiviral drugs and antibiotics are commonly used to treat ARIs in clinic, however, increasing drug resistance, toxicity, side effects, are formidable problems [4]. Therefore, alternative effective modalities for the treatment of respiratory infections are needed. And there is a growing recognition of the herbal medicine as its effectiveness has been investigated scientifically and a long history of native medicine [5].

**Fig. 1:**
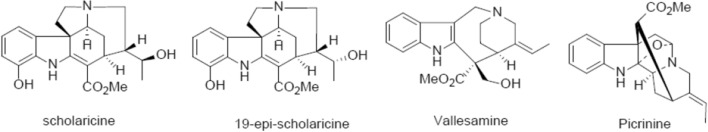
Four major alkaloids from *A. scholaris*

*Alstonia scholaris* (L.) R. Br., an evergreen tree of Apocynaceae, is widely distributed in southwestern China, India, Thailand, Malaysia, Philippines, Africa, Australia, etc. [6]. In traditional medicine, the leaf of *A. scholaris* has been also used for treatment of many acute and chronic respiratory disorders for centuries [7–10]. The total alkaloids extract (TA) was obtained from the leaves of *A. scholaris* by extraction with ethyl acetate, and indole alkaloid content was more than 50%. Various laboratory-identified biological activities have been reported by our research group, including antitussive, anti-asthmatic, expectorant [11], analgesic, anti-inflammatory [12], anti-airway inflammation [13], anti-allergic asthma [14], anti-postinfectious cough [15], alleviating emphysema [16] and pulmonary fibrosis [17]. The anti-inflammatory effects were achieved by triggering the β_2_ adrenergic receptor [18] and inhibiting the nuclear factor-κB expression [19]. In addition, preclinical safety evaluation results indicated that TA had a wide safety range in rats [20] or dogs [21] and no genotoxic effects [22]. Notably, the human pharmacokinetic results [23] were consistent with those of preclinical studies [14, 24], and the four principal alkaloids (picrinine, scholaricine, vallesamine, and 19-epischolaricine, Fig. 1) were all distributed in blood, thus ensuring credibility and validity for further study.

In addition, the chemical components of different parts of the herb were intensively investigated by our group [25–45]. In the leaves of this plant, picrinine, scholaricine, vallesamine, and 19-epischolaricine were proved to be the major bioactive alkaloids. TA extracted from *A. scholaris* leaves was approved as a new botanical drug (No. 2011L01436) by the China Food and Drug Administration, due to its strong pharmacological activity, low toxicity, and stable extraction process. Herein, we described antiviral (RSV, HSV-1, H1N1) and anti-bacterial (beta-hemolytic *streptococcus*) property in vitro and in vivo of TA*,* which might cause acute respiratory infections clinically.

## Results

### Virus Infectivity

As shown in Table S1, the CPE of RSV and HSV-1 on Hep 2 and Vero cells at 10^–13^, 10^–12^, 10^–11^ and 10^–10^ groups were 25–70% of normal group, suggesting the RSV and HSV-1 preparations met the virulence criteria for a successful model of HSV-1 infection. The TCID_50_ of RSV and HSV-1 were 10^–12^ and 10^–11^, respectively. In subsequent experiments, 100 folds TCID_50_/mL of RSV and 10 folds TCID_50_/mL of HSV-1 solution were selected.

### The Effect of TA on Cell Viability

As shown in Table S2, the cell viability of TA on Hep 2 and Vero cells was pretty much the same, the TC_50_ value and TC_0_ was 18.75 and 6.25 mg/mL, respectively. They were all 1.25 (TC_50_) and 0.313 (TC_0_) mg/mL for ribavirinc and acyclovird.

### TA Inhibited the RSV and HSV-1 Virus Replication In Vitro

The antiviral activity of TA extracted from *A. scholaris* was evaluated against RSV and HSV-1 by CPE inhibition assay (Table 1). Compared with control group, nearly all cells in RSV and HSV-1 alone group were dead (data not shown). Compared with RSV and HSV-1 alone group, TA exhibited satisfied anti-RSV and anti-HSV-1 activity, the un-infected cells in TA groups was significantly increased in a dose-dependent manner, and the IC_50_ value was 3.13 and 1.56 mg/mL, the TI was 6 and 12, respectively. Positive control, ribavirinc and acyclovird also exhibited strong antiviral activity, with IC_50_ values ranging from 0.02 (ribavirinc) to 0.01 mg/mL (acyclovird), and the TI value was 62.5 and 125, respectively.

**Table 1:** The inhibitory effect of TA against RSV and HSV-1 viruses in vitro

Conc^a^	RSV			HSV-1		
	TA^b^	Conc^c^	Ribavirinc^d^	TA^e^	Conc^f^	Acyclovird^g^
6.25	75	0.625	ND	100	0.625	ND
3.13	50	0.313	100	75	0.313	100
1.56	25	0.156	100	50	0.156	100
0.78	0	0.078	100	25	0.078	100
0.39	0	0.039	75	0	0.039	100
0.2	0	0.02	50	0	0.02	75
0.1	0	0.01	25	0	0.01	50
0.05	ND	0.005	0	ND	0.005	25

Ribavirinc and Acyclovird were the positive control

*Conc* concentration, *RSV* respiratory syncytial virus, *HSV-1* herpes simplex virus type 1, *ND* not done

^a^The concentration of TA (mg/mL)

^b^The percentage of Hep 2 cells not infected by RSV viruses of TA (%)

^c^The concentration of ribavirinc (mg/mL)

^d^The percentage of Hep 2 cells not infected by RSV viruses of ribavirinc (%)

^e^The percentage of Vero cells not infected by HSV-1 viruses of TA (%)

^f^The concentration of acyclovird (mg/mL)

^g^The percentage of Vero cells not infected by HSV-1 viruses of acyclovird (%)

### TA Inhibited H1N1 Replication In Vitro

The chicken embryos were treated with TA in a dose-dependent manner to determine HI. As shown in Table 2, strong anti-H1N1 activity of TA was observed, with HI values (50 mg/mL: 1/160; 25 mg/mL: 1/320; 12.5 mg/mL: 1/480, respectively) comparable to that of control (Kugan granule, 1/160).

**Table 2:** The inhibitory effect of TA against H1N1 virus in vitro

Group	HI	Dilution	Results
Normal	0	–	–
Model	1/1280	0	–
Control	1/160	3	significant
TA-50	1/160	3	significant
TA-25	1/320	2	significant
TA-12.5	1/480	1.5	effective

HI: Hemagglutination titer

TA was dosed from 12.5 mg/kg to 50 mg/kg. The virus titer of H1N1 declined by one dilution was considered to be effective, and significant effect by two dilutions, when compared with the model group

### TA Blocked H1N1-Infection in Mice

To further study the ability of TA to block H1N1 infectivity in vivo, we used a well-known mouse model of nasal H1N1 infection. Table S3 showed the LD_50_ of H1N1 was 1/200. Consequently, for the following experiments, mice were intranasally infected with 10 times LD_50_ of the virus. As shown in Table 3, infection with 10 times LD_50_ of H1N1 led to the death of all mice of model group within 14 days. Mice showed signs of piloerection, lethargy, weight loss and reduced food intake 3 days after H1N1 inoculation, and some of them died within 7–10 days of infection. Table 3 showed that the survival rate was 0% in the virus model group, but increased to 73.3%, 76.7% and 66.7% on 14 d in 50, 25 and 12.5 mg/kg of TA groups, respectively.

**Table 3:** The inhibitory effect of TA against H1N1 virus in vivo

Group	N	Survivors	Survival rate (%)	Mean survival time	Elongation ratio (%)	Lung weights (g)	Inhibition ratio (%)
Normal	30	30	–	14 ± 0.0	–	0.17 ± 0.016	–
Model	30	0	–	7.00 ± 2.30^▲▲^	–	0.30 ± 0.035^▲▲^	–
Control	30	20	66.7*	12.03 ± 2.94**	71.9	0.21 ± 0.017*	69.2
TA-50	30	22	73.3*	12.27 ± 3.02**	75.3	0.21 ± 0.015*	69.2
TA-25	30	23	76.7*	12.37 ± 3.17**	76.7	0.22 ± 0.015*	61.5
TA-12.5	30	20	66.7*	11.63 ± 3.61**	66.1	0.23 ± 0.015*	53.9

Values represent the mean ± standard deviations (SD) of three independent experiments for anti-H1N1 assay. TA was dosed from 12.5 mg/kg to 50 mg/kg. ^▲▲^*p* < 0.01 *vs.* the normal group; ^*^p < 0.05 or ^**^p < 0.01 *vs.* the model group

Meantime, the mean survival time of model group was 7.0 ± 2.3 days only, whereas, it was significantly prolonged to 12.27 ± 3.02, 12.37 ± 3.17 and 11.63 ± 3.61 days (all p < 0.01), the elongation ratio increased by 75.3%, 76.7 and 66.1% after the oral intervention of TA (50, 25 and 12.5 mg/kg).

In addition, weight of lung tissues was determined to verify whether TA could protect against H1N1 induced pulmonary injury. The lung weight in the model group (0.30 ± 0.035 g) was increased compared to the normal group (0.17 ± 0.016 g), indicating that H1N1 infection could cause swelling of lung tissues. Expectedly, it was showed a significant decrease of lung tissue weight in three doses of TA, and the average weights of lungs were 0.21 ± 0.015 g, 0.22 ± 0.015 g, and 0.23 ± 0.015 g compared with the virus-infected model group (0.30 ± 0.035 g, p < 0.01). Notably, the inhibitory effect of TA was comparable to control. Therefore, the minimum effective dose (MED) of TA against influenza A virus in Balb/C mice was 12.5 mg/kg and the TI was 64.

### Protective Effect of TA Against Mice Infected with Beta-Hemolytic *Streptococcus*

As shown in Table S4, the MLD of beta-hemolytic *streptococcus* was determine to be 1 × 10^9^ CFUs on female KM mice. Thus, for the follow-up experiments, mice were intraperitoneally infected with MLD of the pathogen.

Survivors of mice were recorded daily for 7 days post-challenge and shown as percentage of animal’s survival (Table 4). The mortality of model group was 100%, while treatment with TA increased the survival from 0 to 40% at all does (400, 200, 100, 50, 25, 12.5 mg/kg), in a non-dose dependent manner, and the ED_50_ value was 83.291 mg/kg. Hence, the TI value was calculated as 65.793 by the formula. Amoxicillin, the standard drug used in this experiment, reduced mice mortality by 0%.

**Table 4:** Protective effect of TA against mice infected with beta-hemolytic *streptococcus*

Group	N	Day 4	Day 5	Day 6	Day 7	Survival rate (%)
Normal	20	0	0	0	0	100
Model	20	11	8	1	0	0
Amoxicillin -300	20	0	0	0	0	100
TA-400	20	8	3	1	0	40
TA-200	20	11	2	0	0	35
TA-100	20	12	3	0	0	25
TA-50	20	16	2	0	0	10
TA-25	20	14	3	0	0	15
TA-12.5	20	17	1	0	0	10

N: Total number of animals in three experiments

Amoxicillin as the positive control. The dose of amoxicillin was 300 mg/kg, and TA was dosed from 12.5 mg/kg to 400 mg/kg

## Discussion

TA, an alkaloids extract from leaves of *A. scholaris*, commonly was used for treating the acute upper respiratory infection in traditional Chinese medicine. It was shown to reduce the numbers of neutrophil and leukocytes, induced by lipopolysaccharide infection and significantly attenuate histopathological changes and excessive secretion of inflammatory cytokines in an airway inflammation rat model.

In present paper, we reported the anti-infective activities of TA that may provide effective therapeutic approaches to the treatment of diseases such as infections of herpes simplex virus (HSV), respiratory syncytial virus (RSV) and influenza A/PR/8/34 (H1N1) virus. HSV, a double stranded DNA virus belonging to the herpesviridae family, involves two human antigen types, HSV-1 and HSV-2 [46]. Clinical manifestations of this viral infection range from benign to severe and life-threating syndromes in immunocompromised patients. In present study, the inhibitory effects of TA against HSV-1 were manifested as a marked reduction in cytopathic effect of Vero, the IC_50_ and TI value were 1.56 mg/mL and 12.

Human respiratory syncytial virus (RSV), an *Orthopneumovirus* belonging to the *Pneumoviridae* family, is one of the most important pathogens causing severe acute lower respiratory infections [47]. The clinical manifestations after RSV infection range from a mild upper respiratory tract infection to severe life-threatening lower respiratory tract involvement such as bronchiolitis, pneumonia, and croup, together with some common symptoms including fever, rhinorrhea, cough, and wheezing [48], which are not readily distinguished from those of other common respiratory virus infections. As expected, the replication of RSV was inhibited by the intervention of TA in vitro, the IC_50_ and TI against RSV were 3.13 mg/mL and 6, respectively.

Influenza A virus, one of the most infectious influenza viruses, contains an eight-segmented negative sensed RNA that codes a total of 14 different proteins [49]. These viral proteins facilitate the virus replication inside the target cell, exploit the host immune system and intracellular pathway store plicate and prompt the releasing of newly formed viral particles from the infected cell [50]. It has been reported that the fatal consequence of the influenza is eminently associated with acute pneumonia which could destabilize the atherosclerotic plaque in the arteries, increasing the risk of heart attack and stroke [51]. The H1N1 virus replication was greatly suppressed in the presence of TA, and the hemagglutination titer was significantly reduced from 1/1280 to 1/160.

In vivo testing without doubt is one of the recognized, if not the most important, essential links between in vitro sensitivity testing and clinical studies. In fact, several guide lines for the clinical evaluation of efficacy and toxicity of anti-infective drugs explicitly require experimental evaluation of new compounds (or novel combinations or therapeutic modalities) in animals as prerequisites for clinical trials [52, 53]. Following, we investigated the protective effect of TA against the H1N1 virus in a mice model by intranasal inoculation virus suspension. Importantly, oral administration of 50, 25, 12.5 mg/kg TA possessed effective antiviral activity in infected-mice, conferring 73.3%, 76.7% and 66.7% protection from death against H1N1, which was superior to those of Kugan granule (66.7%). TA also prolonged survival time and decreased lung weights in virus-infected mice. Taken together, the antiviral effects of TA against influenza virus were demonstrated in this study, which agrees with findings of previous studies [54].

Moreover, upper respiratory infection caused by virus can easily lead to secondary infections caused by bacteria. Beta-hemolytic *streptococcus* is one of the major human pathogens that cause both community- and hospital-acquired infections [55]. The infections caused by it ranged from the relatively mild superficial skin purulent inflammation to the more serious conditions as respiratory tract infection, epidemic pharyngitis, neonatal sepsis, bacterial endocarditis, scarlet fever, rheumatic fever and glomerulonephritis [56]. First of all, the upper respiratory tract mucous membrane was damaged by beta-hemolytic *streptococcus* and its discharge toxins, with the symptoms of pharyngeal hyperemia, edema. Furtherly, local inflammatory cell infiltration and seepage resulted in redness and swelling of the pharynx, tonsil, soft palate and even uvula. Eventually, tonsil and soft palate mucosa often undergo necrosis and develop into purulent pharyngeal buccal inflammation and tonsils.

Dose bacteria pathogenicity experiments were conducted to determine the MLD of the pathogen beta-hemolytic *streptococcus* infection in mice. The MLD_50_ was 1 × 10^9^ CFUs in female mice. Therefore, for subsequent experiments, mice were infected with MLD of the pathogen by intraperitoneal injection. In particular, TA, at all the doses tested, effectively protected the mice from infected by beta-hemolytic *streptococcus* and increased animal survival rate, thus establishing the potential in vivo antibacterial activity of TA and further supporting its traditional use in the treatments of ARIs.

## Materials and Methods

### Plant Materials

Leaves of *A. scholaris* were purchased from Datang–Hanfang Medicine Co., Ltd. (Pu’er, China). The plants were collected in 2006 in Pu’er City, Yunnan Province, People’s Republic of China. Dr. Xiao–Dong Luo of the Kunming Institute of Botany, Chinese Academy of Sciences, identified the plants, and the plant name was checked against http://www.theplantlist.org. A voucher specimen (no. Luo20060407) was deposited in the State Key Laboratory of Phytochemistry and Plant Resources in West China, Chinese Academy of Sciences.

### Preparation of Alkaloids

Dried and powdered leaves of *A. scholaris* were extracted with 90% ethyl alcohol under reflux conditions (3 h × 4 times) at room temperature, and the solvent was evaporated *in vacuo* to obtain the ethanolic extract. The ethanolic extract was dissolved in 0.3% aqueous HCl solution and filtered; the residue was the nonalkaloid fraction. Then, the acidic solution, adjusted to pH 9–10 with 10% aqueous ammonia, was extracted with ethyl acetate to obtain the total alkaloids (TA) fraction (batch no. 20070512, 20111101).

### Chemicals, Viruses, Bacteria and Cells

Acyclovir and ribavirin were selected to be antiviral control against HSV-1 and RSV and provided by Tianjin Pharmaceutical Jiaozuo Co, Ltd (Tianjin, China). Kugan granule, a launched traditional Chinese medicine formula, was used to be antiviral control against H1N1, and provided by Qingdao Guofeng Pharmaceutical Co. Ltd (Qingdao, Shandong). Amoxicillin was chosen as an antibacterial control and produced by Zhuhai Federal Pharmaceutical Co. Ltd (Hongkong, China).

The influenza strains A/FM1/1/47 (H1N1) and herpes simplex virus type 1 strain sm44 (HSV-1) were obtained from National Institute for Viral Disease Control and Prevention, China Center for Disease Control and Prevention. Respiratory Syncytial Virus (RSV) was provided by West China School of Basic Medical Sciences & Forensic Medicine, Sichuan University. Hep2 and Vero cells were purchased from Conservation Genetics CAS Shanghai Cell Bank. Specific-pathogen-free embryonated eggs were denoted by West China School of Basic Medical Sciences & Forensic Medicine, Sichuan University. Standard strain beta-hemolytic *streptococcus* was purchased from the American Type Culture Collection (ATCC, Rockville, MD, USA).

### Animals

Specific-pathogen-free, female, Balb/C and KM mice (weight: approximately 20 g) were purchased from West China Medical Laboratory Animal Center, Sichuan University (license number SCXK [Chuan] 3-042-2010). Our experiments were approved by the Institutional Animal Care and Use Committee of the Sichuan University and performed according to the international rules concerning animal experiments and the internationally accepted ethical principles for the use and care of laboratory animals.

### Virus Virulence and Cytotoxicity Assay

The anti-RSV and anti-HSV-1 activities of TA were determined by the titer reduction assay as previously described [57]. Briefly, Hep2 (to RSV) and Vero cell monolayers (to HSV-1) were co-incubated with a series of virus concentrations from 10^–3^ to 10^–13^. The cytopathogenic effect (CPE) was observed and half of tissue culture infective dose (TCID_50_) was calculated according to Reed & Muench formula [58].

The half toxicity (TC_50_) and non-cytotoxic concentration (TC_0_) of TA against Hep2 and Vero cells were determined using a 3-(4,5-dimethylthiazol-2-yl)-2,5-diphenyltetrazolium bromide assay as previous [59].

### Anti- HSV-1 and—RSV Activity Assay In Vitro

Methods was referred to the previous with minor modifications [60], TA was suspended in 1% dimethylsulfoxide/sterile water for injection. Equivalent of 100 folds TCID_50_/mL of RSV for Hep2 cells and 10 folds TCID_50_/mL of HSV-1 for Vero cells were added to the cell cultures which were then incubated at 37 °C in a 5% CO2 atmosphere, and the supernatant was removed after incubation for 2 h. Cells were then treated with serial concentrations of TA diluted from TC_50_ to 1/64 TC_50_ (mg/mL) or with two-fold serial dilutions of ribavirin and acyclovir (from 1/2 TC_50_ to 1/128 TC_50_) used as the positive control. All wells were then observed under a light microscope to determine the virus-induced cytopathic effect (CPE) at day 5 after the treatment. The concentrations of the test articles that reduced CPE by 50% (IC_50_) were determined. And the therapeutic index (TI) value was calculated as the ratio TC_50_/IC_50_ [61].

### Effect of TA on H1N1-Infection In Vitro

The efficacy of TA against H1N1 virus in vitro was studied by cultivation of chicken embryo and haemagglutination inhibition test as previously reported [62]. The proliferative activity of influenza virus H1N1 was determined by chicken embryo method and the virus titers was 1/1280. All embryonated eggs except the normal group were injected with 0.1 mL H1N1 virus (60 U/mL) via allantoic cavity inoculation before treatment. Then the eggs inoculated virus were randomly divided into 5 groups. The embryonated eggs in model group received 0.1 mL of 1% DMSO, the embryonated eggs in the positive group received 0.1 mL of control; group 3 to 5 were administrated different concentrations of TA treatment, and finally, the normal group was also inoculated with 0.1 mL of 1% DMSO. The concentration of TA was non-toxic to chicken embryos confirmed by a preliminary experiment (data not shown). Allantoic fluid of chicken embryo were collected from each group at 48 h post infection, and the hemagglutination titer (HI) was determined by hemagglutination assay with 1% chicken red blood cell. The virus titer of H1N1 declined by one dilution was considered to be effective, and significant effect by two dilutions, when compared the model group.

### Effect of TA on H1N1-Infection in Mice

A preliminary examination was carried out before the formal experiment began to determine the virulence of H1N1 virus on mice. For infectious experiment, 60 eight weeks BALB/c female mice were divided into six groups including normal (mice without viral infection); model (infected mice); control (Kugan granule, 960 mg/kg) and TA-treated groups (50, 25, 12.5 mg/kg). The choice of the doses used in this experiment was based on the pilot study [63] and the results from acute oral toxicity study [20]. The treatment started 2 days before viral challenge. Accordingly, treatment of the mice with TA or positive control started two days before infectious. For the normal and model group, the mice were given 1% DMSO instead. Subsequently, all mice except the normal group were challenged with 10 times LD_50_ of H1N1 by intranasal inoculation of 30 μL of virus suspension. An equal volume of intranasal saline was substituted for the normal group. Following viral, the treatment was repeated daily for 14 consecutive days and survival animals were recorded for 14 days, and the lungs were then collected and weighted.

### Anti-Beta-Hemolytic *Streptococcus* Assay in Mice

The minimum lethal dose (MLD) was regarded as the minimum amount of infectious bacteria that caused all the infected animals to die within 1–2 days after the intraperitoneal injection of beta-hemolytic *streptococcus* suspension [64]. Briefly, forty-five female KM mice were randomly divided into nine groups (each group of five). Then, forty animals in different groups were administrated intraperitoneally with beta-hemolytic *streptococcus* suspension (10^10^–10^3^/mL) at a volume of 0.5 mL/mouse. Normal animals were given the same volume of saline solution.

As for the protection against beta-hemolytic *streptococcus *in vivo, the mice were divided into nine groups of ten mice each: normal (mice without bacterial infection); model (infected mice); amoxicillin-treated group (positive control, 300 mg/kg) and six TA-treated groups (400, 200, 100, 50, 25, 12.5 mg/kg). The oral treatment was administered two days prior to infection. For normal and model groups, 1% DMSO was administered by oral gavage. Subsequently, the bacteria-infected groups except normal group were inoculated intraperitoneally with MLD of 0.5 mL of beta-hemolytic *streptococcus* suspension. For the normal group, the mice were given the same volume saline water in place of the bacterial suspension. Following bacterial challenge, the treatment was repeated one time after infectious. The clinical signs and surviving animals were recorded daily till 7 days post-infection. Half of the effective dose (ED_50_) was calculated using Bliss method, and then the therapeutic index (TI) at each test was determined using the following formula: TI = LD_50_/ED_50_ (LD_50_: half of the lethal dose, 5.48 g/kg).

### Statistical Analysis

Data from the experiment are expressed as the mean ± standard deviation of the mean. Two-tailed Mann–Whitney U test was used for comparing between two groups, one-way analysis of variance with least significant difference or Kruskal–Wallis analysis with Dunn post-test for multiple comparisons and chi-square test, in which nonparametric analyses were appropriate. Analyses were performed using the Prism software (GraphPad, San Diego, CA, USA). A *p*-value < 0.05 denoted statistical significance in all analyses.

## Supplementary information

Below is the link to the electronic supplementary material.

Supplementary information 1 (DOCX 18 kb)

## Abbreviations

TA: Total alkaloids extract
ARIs: Acute respiratory infections
H1N1: Influenza strains A/FM1/1/47
HSV-1: Herpes simplex virus type 1 strain sm44
RSV: Respiratory syncytial virus
IC_50_: Half inhibitory concentration
TI: Therapeutic index
CPE: Cytopathogenic effect
TCID_50_: Half of tissue culture infective dose
TC_50_: Half toxicity
TC_0_: Non-cytotoxic concentration
DMSO: Dimethyl sulfoxide
MLD: The minimum lethal dose
ED_50_: Half of the effective dose
LD_50_: Half of the lethal dose
RPMI: Roswell Park Memorial Institute
FBS: Fetal bovine serum
MLD: The minimum lethal dose
MED: The minimum effective dose
