# Supplementary material for: Anti-microbial Effects In Vitro and In Vivo of Alstonia scholaris
Source: Nat Prod Bioprospect. 2021 Jan 3;11(1):127–35. doi: 10.1007/s13659-020-00294-6 (PMC7778864; doi:10.1007/s13659-020-00294-6)
Supplement: Supplementary file 1 — Supplementary information 1 (DOCX 18 kb) [file 13659_2020_294_MOESM1_ESM.docx]

**Supporting Information for**

Yun-Li Zhao ^a,1^, Zhong-Ping Gou ^c,1^, Jian-Hua Shang ^b^, Wan-Yi Li ^d^, Yu Kuang ^d^, Ming-Yuan Li ^d,^*, Xiao-Dong Luo ^a,b,^*

^a^ *Key Laboratory of Medicinal Chemistry for Natural Resource, Ministry of Education and Yunnan Province, School of Chemical Science and Technology, Yunnan University, Kunming 650091, People’s Republic of China*

*^b^ State Key Laboratory of Phytochemistry and Plant Resources in West China, Kunming Institute of Botany, Chinese Academy of Sciences, Kunming 650201, P. R. China*

*^c^* *Institute of Drug Clinical Trials, West China Hospital, Sichuan University, Chengdu, 610041, China*

*^d^* *West China School of Basic Medical Sciences & Forensic Medicine, Sichuan University, Chengdu, 610041, China*

__________________________________________________

*** Corresponding author. Tel.: +86 871 65223177; fax: +86 871 65220227.

*E-mail address:* [xdluo@mail.kib.ac.cn](mailto:xdluo@mail.kib.ac.cn) (X.-D. Luo); [lmy3985@sina.com](mailto:lmy3985@sina.com) (M.-Y. Li)

^1^These authors contributed equally.

**Table S1 The cytopathic effect (CPE) of virus on cells**

| Viral titers (1×10^X^) | RSV^a^ | HSV-1^b^ |
| --- | --- | --- |
| Normal | 0 | 0 |
| -3 | 100 | 100 |
| -4 | 100 | 100 |
| -5 | 100 | 100 |
| -6 | 100 | 100 |
| -7 | 100 | 100 |
| -8 | 100 | 100 |
| -9 | 100 | 100 |
| -10 | 75 | 75 |
| -11 | 75 | 50 |
| -12 | 50 | 25 |
| -13 | 25 | ND |

ND: Not done

a: The CPE value of RSV on Hep 2 cells.

b: The CPE value of HSV-1 on Vero cells.

**Table S2 The effect of TA, ribavirin and acyclovir on cell viability**

| Conc^a^ | TA^b^ | Conc^c^ | Ribavirinc^d^ | Acyclovird^e^ |
| --- | --- | --- | --- | --- |
| 50 | 0 | 5 | 0 | 0 |
| 25 | 25 | 2.5 | 25 | 0 |
| 12.5 | 75 | 1.25 | 50 | 50 |
| 6.25 | 0 | 0.625 | 75 | 75 |
| 3.13 | 0 | 0.313 | 0 | 0 |
| 1.56 | 0 | 0.156 | 0 | 0 |
| 0.78 | 0 | 0.078 | 0 | 0 |

Conc: Concentration

^a^: The concentration of TA (mg/mL).

^b^: The viability of TA on Hep 2 and Vero cells (%).

^c^: The concentration of Ribavirinc and Acyclovird (mg/mL).

^d^: The viability of Ribavirinc on Hep 2 cells (%).

^e^: The viability of Acyclovird (%) on Vero cells.

**Table S3 The virulence of H1N1 on mice**

| Virus titer | N | Mortality (%) |
| --- | --- | --- |
| Normal | 10 | 0 |
| 1/50 | 10 | 100 |
| 1/100 | 10 | 80 |
| 1/200 | 10 | 60 |
| 1/400 | 10 | 20 |
| 1/800 | 10 | 0 |

N: The numbers of mice in each group.

Normal: Mice were intranasally inoculation with saline solution.

**Table S4 The mortality of mice infected with beta-hemolytic *streptococcus***

| Conc (1×10^X^ CFU/mL) | N | Day 1 | Day 2 | Mortality (%) |
| --- | --- | --- | --- | --- |
| Normal | 5 | 0 | 0 | 0 |
| 10 | 5 | 4 | 1 | 100 |
| 9 | 5 | 2 | 3 | 100 |
| 8 | 5 | 2 | 2 | 80 |
| 7 | 5 | 0 | 2 | 40 |
| 6 | 5 | 0 | 0 | 0 |
| 5 | 5 | 0 | 0 | 0 |
| 4 | 5 | 0 | 0 | 0 |
| 3 | 5 | 0 | 0 | 0 |

Conc: The concentration of beta-hemolytic *streptococcus suspension.*

N: The numbers of mice in each group.

Normal: Mice were intraperitoneally injected with saline solution.
